# DHA Suppresses Primary Macrophage Inflammatory Responses via Notch 1/ Jagged 1 Signaling

**DOI:** 10.1038/srep22276

**Published:** 2016-03-04

**Authors:** Mehboob Ali, Kathryn Heyob, Lynette K. Rogers

**Affiliations:** 1 Center for Perinatal Research, The Research Institute at Nationwide Children’s Hospital, Columbus, Ohio, USA; 2 Department of Pediatrics, The Ohio State University, Columbus, Ohio, USA

## Abstract

Persistent macrophages were observed in the lungs of murine offspring exposed to maternal LPS and neonatal hyperoxia. Maternal docosahexaenoic acid (DHA) supplementation prevented the accumulation of macrophages and improved lung development. We hypothesized that these macrophages are responsible for pathologies observed in this model and the effects of DHA supplementation. Primary macrophages were isolated from adult mice fed standard chow, control diets, or DHA supplemented diets. Macrophages were exposed to hyperoxia (O_2_) for 24 h and LPS for 6 h or 24 h. Our data demonstrate significant attenuation of Notch 1 and Jagged 1 protein levels in response to DHA supplementation *in vivo* but similar results were not evident in macrophages isolated from mice fed standard chow and supplemented with DHA *in vitro*. Co-culture of activated macrophages with MLE12 epithelial cells resulted in the release of high mobility group box 1 and leukotriene B_4_ from the epithelial cells and this release was attenuated by DHA supplementation. Collectively, our data indicate that long term supplementation with DHA as observed *in vivo*, resulted in deceased Notch 1/Jagged 1 protein expression however, DHA supplementation *in vitro* was sufficient to suppress release LTB_4_ and to protect epithelial cells in co-culture.

Docosahexaenoic acid (DHA) is an omega-3 long chain fatty acid (LCFA) that is an effective natural product for attenuation of inflammation in many diseases processes^1^,^2^. In the context of acute inflammation such as lipopolysaccharide (LPS) exposure, LCFAs inhibit toll-like receptor (TLR) signaling and thus inhibit NFkB-mediated pathways, specifically in macrophages^3^,^4^. Others have speculated that DHA-mediated changes in membrane fluidity and lipid raft composition are responsible for altered receptor presentation, possibly through interfering with dimerization, and decreased signaling^5^,^6^.

In our murine model of perinatal inflammation, we previously observed sustained increases in macrophage numbers, even in adulthood, in the mice exposed to prenatal LPS and postnatal hyperoxia^7^,^8^. Additionally, we observed that feeding the pregnant dam a diet supplemented with docosahexaenoic acid (DHA) prior to LPS exposure and during nursing and hyperoxia exposure, decreased the number of macrophages found in the lungs of the pups^9^. While the role of these persistent macrophages in pathogenesis hyperoxia-induced lung disease is unknown, we speculate that they are partly responsible for ongoing lung tissue remodeling and apoptosis observed in this model^10^. Furthermore, we speculate that dietary DHA supplementation is altering receptor presentation and/or signaling to dampen inflammatory responses^5^. Dietary supplementation for a period of time will allow DHA to be incorporated into membrane phospholipids while shorter exposures may have direct impact on signaling pathways.

Macrophages accumulate in response to inflammation and facilitate host defense^11^. Previous reports have shown that bacterial infection as well as hyperoxia exposure can alter macrophage function in the lungs resulting in prolonged or aberrant release of injurious substances and propagation of further injury to adjacent lung cells^12^,^13^. Further, DHA supplementation has been shown to shift macrophage phenotype to M2 responses and facilitate resolution^14^,^15^,^16^. Our question was whether changes in macrophage phenotype in our adult offspring previously exposed to perinatal inflammation were responsible for the exacerbated and prolonged pathologies observed in our model.

Notch signaling is essential for normal lung growth and development and inflammation and hyperoxia have been reported to alter Notch pathways^17^,^18^. Our recent publication investigated Notch signaling in whole lung homogenates from mice exposed to prenatal LPS and neonatal hyperoxia^10^. While we did not observe consistent differences in Notch pathway proteins, we did observe trends toward changes in Notch signaling in our model, suggesting that the alterations in signaling may be occurring in a single cell type and not readily observable in whole lung preparations. Furthermore, others have reported that Notch signaling favors M1 polarization and a pro-inflammatory macrophage phenotype which could be responsible for release of substances injurious to adjacent cells^17^,^19^,^20^ while DHA favors M2 polarization^14^,^15^,^16^. High mobility group box 1 (HMGB1) and leukotriene B_4_ (LTB_4_) are potent mediators released from macrophages in response to LPS but their role in macrophage-induced epithelial injury and dysfunction or their relation with Notch signaling has not been extensively explored^21^.

In the present study, we tested the hypothesis that DHA supplementation *in vivo*, using diets enriched in DHA, or *in vitro*, using direct DHA administration, would attenuate the effects of combined LPS and hyperoxia exposure on lung primary macrophages and immortalized MHS cells. To accomplish this we investigated the effects of DHA on antioxidant capacity, Notch expression, apoptosis, and the release of injurious mediators in co-cultured epithelial cells.

## Results

### Glutathione related antioxidants

Oxidation was assessed by measuring glutathione (GSH), glutathione disulfide (GSSG), glutathione reductase (GR), and glutathione peroxidase (GPX) in primary macrophages treated with DHA *in vivo* and *in vitro* (Table 1). While DHA supplementation substantially increased GSH contents in room air (RA, 21% O_2_)/phosphate buffered saline (PBS) treated macrophages compared to macrophages from controls, differences in other treatment groups were modest. Similarly, GSSG contents were elevated in the RA/PBS treatment group by DHA supplementation compared to control but minimal differences were observed with supplementation within the treatment groups. GR activities were elevated by DHA supplementation in the RA/PBS treated groups compared to macrophages from control groups and was elevated due to O_2_ and/or LPS treatments both *in vitro* and *in vivo*. DHA further elevated GR activity in the group supplemented *in vivo* and treated with O_2_ and/or LPS. GPx activity was not affected by DHA or treatments.

### Notch signaling pathways

**Figure 1:**
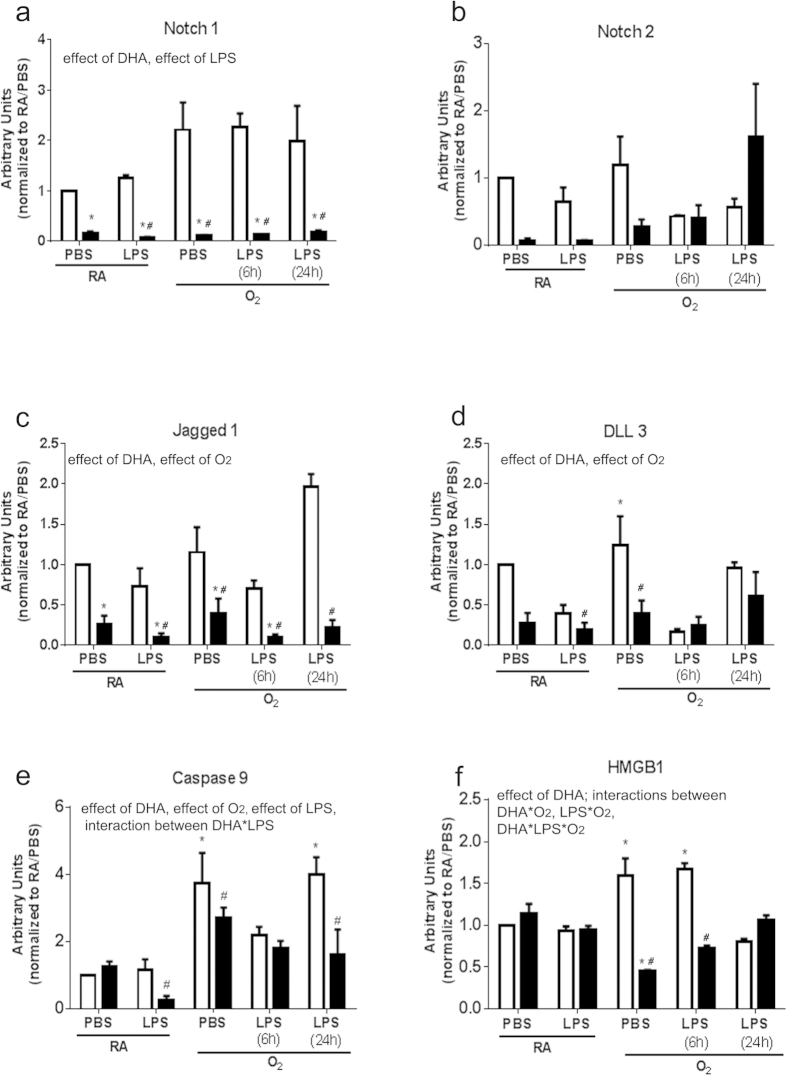
Western blot analyses for Notch pathway (**a–d**), caspase 9 (**e**), and HMGB1 (**f**) proteins were performed on homogenates from primary macrophages isolated from mice fed CD or DHA supplemented diets *(in vivo)* and subsequently treated with O_2_ and/or LPS. Separation was performed by standard protocols as described in Methods and blots were quantified by densitometry. White bars indicate CD, black bars indicate DHA supplemented diets. Data were analyzed by using a Multivariate Linear Regression Models with diet as a fixed factor, treatment and exposure as co-variants, and 2 and 3-way interactions were assessed. Differences within individual groups was analyzed by Tukey's post hoc. The data reflect n = 3 from three independent experiments. Major effects and interactions are indicated on the graphs. Post hoc analyses are indicate by * different than CD-RA/PBS; ^#^ different than same treatment (difference between diets), p < 0.05.

**Figure 2:**
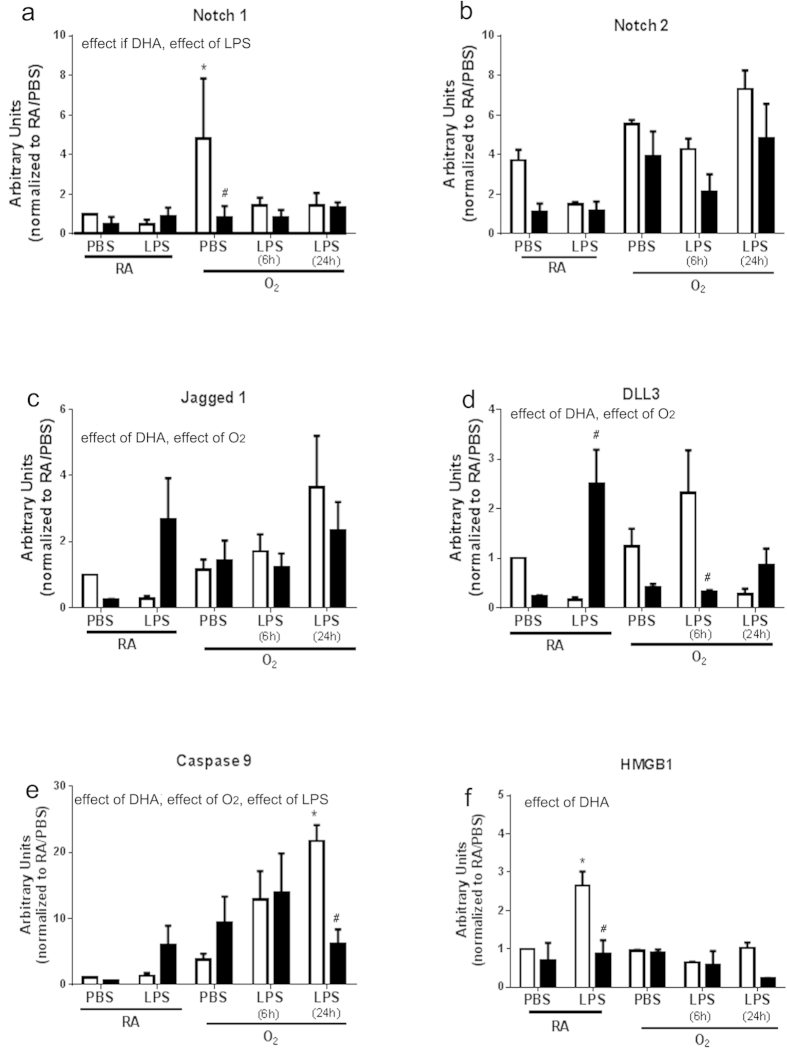
Western blot analyses for Notch pathway (**a–d**), caspase 9 (**e**), and HMGB1 (**f**) proteins were performed on homogenates from primary macrophages isolated from mice fed standard diets, supplemented with vehicle or DHA in culture *(in vitro)*, and subsequently treated with O_2_ and/or LPS. Separation was performed by standard protocols as described in Methods and blots were quantified by densitometry. White bars indicate vehicle, black bars indicate DHA supplement. Data were analyzed by using a Multivariate Linear Regression Models with diet as a fixed factor, treatment and exposure as co-variants, and 2 and 3-way interactions were assessed. Differences within individual groups was analyzed by Tukey's post hoc. The data reflect n = 3 from three independent experiments. Major effects and interactions are indicated on the graphs. Post hoc analyses are indicate by * different than CD-RA/PBS; ^#^ different than same treatment (difference between diets), p < 0.05.

Notch 1 protein levels were increased by LPS treatment compared to PBS in the macrophages isolated from the CD fed mice (Fig. 1a). Macrophages isolated from mice fed DHA *in vivo* exhibited dramatic suppression of Notch 1 protein expression in all treatment groups indicating an effect of DHA. The increase in Notch 1 signaling due to LPS treatment was not as profound in the macrophages isolated from mice fed standard chow and supplemented *in vitro* however, DHA supplementation did suppress Notch 1 expression overall (Fig. 2a) indicating an effect of DHA, and LPS. While Notch 2 expression in macrophages supplemented *in vivo* followed a pattern similar to Notch 1 with the exception of O_2_/LPS at 24 h no statistical differences were indicated (Fig. 1b). Macrophages supplemented with DHA *in vitro* indicated no statistical differences with treatments (Fig. 2b). A pattern of induction similar to Notch 1 was observed in Jagged 1 with increases due to O_2_ exposure compared to RA and decreases in expression associated with DHA supplementation in all treatment groups in macrophages isolated from mice supplemented *in vivo* (Fig. 1c). An effect of DHA and O_2_ were indicated in Jagged 1 expression in the macrophages supplemented with DHA *in vitro* (Fig. 2c). A trend toward DHA-induced decreased DLL 3 expression was observed in macrophages supplemented *in vivo* indicating an effect of DHA and LPS treatment (Fig. 1d). A similar pattern was observed in macrophages supplemented *in vitro* with effects of DHA and O_2_ exposure (Fig. 2d). The Notch pathway proteins NUMB, Jagged 2, Nicast, Presnillin 1 and Presnillin 2 were also measured but no differences were observed (data not shown).

### Assessments of apoptosis

Cell death in primary macrophages treated with O_2_ and LPS was assessed by measuring caspase 9 protein levels. Caspase 9 levels were elevated in the CD-O_2_/PBS and O_2_/LPS (24h) groups compared to control RA/PBS group and DHA supplementation was able to attenuate these increases (Fig. 1e). An effect of DHA, O_2_, and LPS and interactions between DHA and LPS were indicated. Caspase 9 levels were increased by O_2_ and/or LPS treatment with or without DHA supplementation *in vitro* with the exception of O_2_/LPS at 24 h (Fig. 2e). These data indicated an effect of DHA, O_2_, and LPS.

HMGB1 levels in the media were increased in the CD-O_2_/PBS and O_2_/LPS (6h) groups and these increases were attenuated in macrophages supplemented with DHA *in vivo* (Fig. 1f) indicating an effect of DHA, and interactions betweem DHA and O_2_, LPS and O_2_, and a 3-way interaction between DHA, LPS, and O_2_. LPS alone induced a significant increase in HMGB1 release in the macrophages supplemented *in vitro* and this increase was attenuated by DHA indicating an effect of DHA (Fig. 2f).

### Co-culture with MLE12 cells

**Figure 3:**
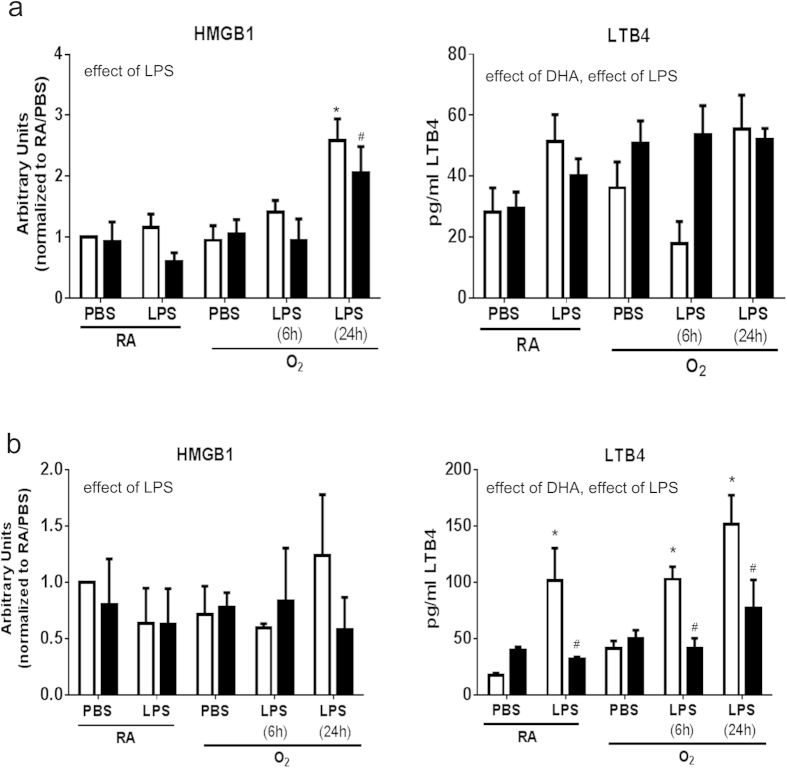
HMGB1 and LTB_4_ were measured in the media of MLE12 cells co-cultured with primary macrophages isolated from mice fed CD or DHA diets *in vivo* (**a**) or mice fed standard diets but supplemented with DHA *in vitro* (**b**). HMGB1 was measured by western blot and LTB_4_ by ELISA. White bars indicate CD or vehicle, black bars indicate DHA supplementation. Data were analyzed by using a Univariate Linear Regression Models with diet as a fixed factor and treatment and exposure as co-variants and 2 and 3-way interactions were assessed. Differences within individual groups was analyzed by Tukey's post hoc. The data reflect n  =  3 from three independent experiments. Major effects and interactions are indicated on the graphs. Post hoc analyses are indicate by * different than CD-RA/PBS; ^#^ different than same treatment (difference between diets), p < 0.05.

Primary macrophages isolated from mice fed CD and DHA supplemented diets were treated with O_2_ and LPS as previously described. After 24 h, the media was removed and the macrophages were placed above confluent MLE12 cells cultured in transwells to identify the effects of DHA on macrophage activation and subsequently on epithelial cell viability. After 24 h, the media from the co-culture was harvested for measurement of HMGB1 and LTB_4_ and the MLE12 cells were harvested and assessed for cl-caspase 3 and Ki67 expression by flow cytometry. The HMGB1 levels were elevated only in the media from MLE12 cells co-cultured with macrophages that were treated with O_2_/LPS for 24 h and DHA supplementation *in vivo* mildly attenuated this increase (Fig. 3a) indicating an effect of LPS. A effect of LPS was observed in the macrophages supplemented with DHA *in vitro* but no individual differences were indicated in post hoc analyses (Fig. 3b). A modest effect of LPS was observed in LTB_4_ release in macrophages treated with O_2_/LPS and an effect of DHA supplementation was observed in the cells supplemented *in vivo* (Fig. 3c). Interestingly, LTB_4_ release was elevated by LPS treatment and this elevation was attenuated by DHA in the cells supplemented *in vitro* indicating an effect of DHA, and LPS (Fig. 3d).

**Figure 4:**
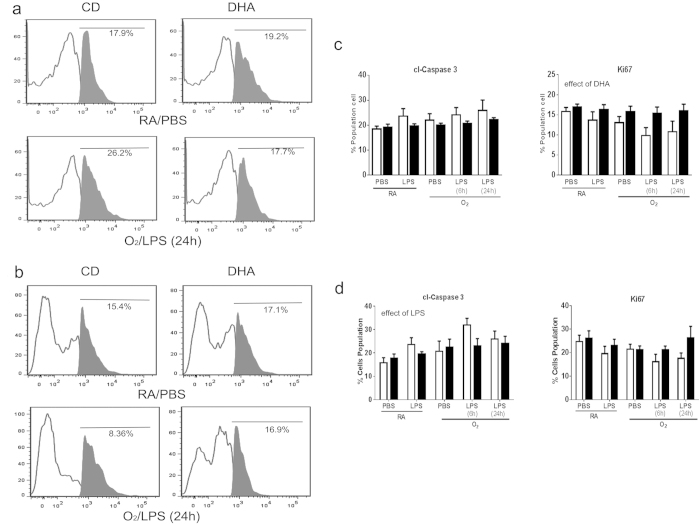
Cl-caspase 3 and Ki67 were measured in MLE12 cells after co-culture with primary macrophages supplemented with DHA *in vivo* (**a, c**) or *in vitro* (**b, d**). Representative examples of flow analyses are presented (**a, b**) and graphs indicating the cumulative results are presented in (**c, d**). White bars indicate CD or vehicle, black bars indicate DHA supplementation. Data were analyzed by using a Univariate Linear Regression Models with diet as a fixed factor and treatment and exposure as co-variants and 2 and 3-way interactions were assessed. Differences within individual groups was analyzed by Tukey's post hoc. The data reflect n = 3 from three independent experiments. Major effects and interactions are indicated on the graphs. Post hoc analyses are indicate by * different than CD-RA/PBS; ^#^ different than same treatment (difference between diets), p < 0.05.

Flow cytometry on the MLE12 cells co-cultured with primary macrophages isolated from mice supplemented with DHA *in vivo* and previously treated with O_2_ and LPS exhibited no change in cl-caspase 3 expression but DHA supplementation preserved proliferation as measured by Ki67 (Fig. 4a,c). MLE12 cells co-cultured with primary macrophages isolated from mice and subsequently supplemented with DHA *in vitro* and treated with O_2_ and LPS exhibited an increase in cl-caspase 3 expression with an effect of LPS but no effect of DHA supplementation. No differences in Ki67 expression were indicated (Fig. 4b,d).

**Figure 5:**
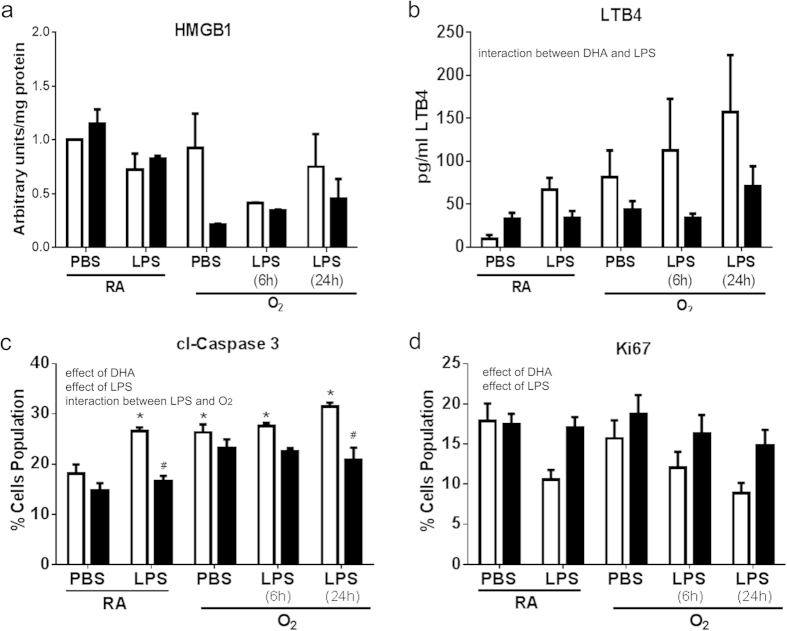
MHS cells were cultured to confluence, supplemented with vehicle or DHA and subsequently exposed to O_2_ and/or LPS. After 24 h, the treated MHS cells were placed in culture with MLE12 epithelial cells. Media was harvested for HMGB1 and LTB4 contents and cells were stained for cl-caspase 3 and Ki67 and analyzed by flow cytometry. Data were analyzed by using a Univariate Linear Regression Models with diet as a fixed factor and treatment and exposure as co-variants and 2 and 3-way interactions were assessed. Differences within individual groups was analyzed by Tukey's post hoc. The data reflect n = 3 from three independent experiments. Major effects and interactions are indicated on the graphs. Post hoc analyses are indicate by * different than CD-RA/PBS; ^#^ different than same treatment (difference between diets), p  <  0.05.

To confirm these findings in pure macrophage populations immortalized mouse macrophage cells (MSH) were cultured on transwell inserts, exposed to O_2_/LPS, and subsequently placed in co-culture with MLE12 cells. There were no statistical differences in HMGB1 levels indicated (Fig. 5a). There was an effect of DHA and LPS with an increase in LTB_4_ release associated with LPS exposure and this increase was attenuated in cells treated with DHA (Fig. 5b). Cl-caspase 3 levels were increased with O_2_ and LPS treatments compared to PBS/RA and the increase was attenuated by DHA supplementation indicating an effect of DHA, LPS, and an interaction between LPS and O_2_ (Fig. 5c). Ki67 levels were decreased with LPS exposure and this decrease was again attenuated by DHA supplementation indicating an effect of DHA and LPS (Fig. 5d).

## Discussion

The combination of maternal inflammation and neonatal hyperoxia results in a severe lung phenotype in newborn C3H/HeN mice with deficits in alveolarization and sustained increases in macrophage numbers in the lungs of the offspring^7^,^8^. Maternal DHA supplementation was able to attenuate the developmental phenotype and decrease lung macrophage numbers in the newborn and older mice^9^. We speculate that the sustained macrophage presence in the lungs of LPS/O_2_-exposed offspring was partly responsible for the severity in developmental deficits and potentially for the ongoing apoptosis observed in this model^10^. Furthermore, we speculate that DHA is exerting anti-inflammatory effects through preventing macrophage activation. In this current study, we tested the hypothesis that DHA supplementation both *in vivo* and *in vitro* would attenuate the effects of combined LPS and hyperoxia exposure on lung primary macrophages. We tested the hypothesis that alterations in antioxidant capacity, Notch signaling, and/or activation of apoptosis pathways were responsible for changes in lung macrophage function and the release of injurious agents that affect adjacent epithelial cells.

Simplistically, macrophages are categorized as M1 pro-inflammatory or as M2 anti-inflammatory^22^. Macrophages are present in the lung mesenchyme early in development and express many of the typical M2 markers^23^. However, inflammation caused by bacteria or sterile stimuli triggers a series of events which includes recruitment of macrophages to the infected/damages tissues and promotes a M1 pro-inflammatory phenotype^24^,^25^,^26^. Recruited macrophages respond by releasing cytokines and the production of reactive oxygen species (ROS) which in turn modulate the anti-oxidant balance. To determine whether DHA supplementation altered this balance in isolated primary macrophages, we measured GSH and GSSG levels as well as the activities of GR and GPx. While minor increases in these antioxidants were evident due to O_2_ and/or LPS treatment and an effect of DHA was observed, there were no clear patterns of increased oxidation or enhanced antioxidant activity (Table 1). This data would imply the DHA supplementation was not altering macrophage signaling through changes in acute oxidant stress as would be indicated by increases in glutathione or glutathione disulfide.

Notch 1 is a cell surface receptor that is essential for many developmental pathways^27^. Notch 1 and its ligand Jagged 1 have been shown to be induced in inflammatory conditions including hyperoxia and LPS exposure^28^. In macrophages, Notch 1 signaling promotes the M1 phenotype and the expression of M1 cytokines^17^,^28^,^29^. Previously, we investigated the effects of neonatal LPS/O_2_ exposure and maternal DHA supplementation on Notch pathway protein expression in whole lung homogenates. Our data indicated modest but statistical differences in Jagged 1, DLL1, NUMB, Presnillin 2, and PEN2 but a trend toward increases in Notch 1 protein expression was evident^30^. We speculated that Notch 1 signaling may be affected in a specific cell type that was not evident in whole lung homogenates. Consequently, we assessed changes in Notch pathway proteins in response to O_2_ and/or LPS exposure in primary macrophages. Furthermore, we tested the hypothesis that DHA supplementation might be attenuating inflammation in this model through modulation of Notch signaling pathways, specifically in macrophages. Our data indicate that indeed Notch 1 and Jagged 1 are both increased with O_2_/ LPS exposure and that this increase is attenuated by DHA supplementation *in vivo* (Fig. 1). We also investigated the effects of DHA supplementation *in vitro* and found that *in vitro* exposure (24 h) did not offer the same attenuation of Notch pathways that was observed in cells isolated from mice supplemented with DHA *in vivo*. There was however, an overall suppression of Notch 1 responses with short term DHA exposure (Fig. 2). Since Notch 1 signaling is linked to apoptotic pathways, caspase 9 was measured in these same cells. Caspase 9 was increased similarly and was normalized by DHA supplementation *in vivo* and in *vitro* at 24 h. These data strongly support the hypothesis that DHA is influencing macrophage function through altering Notch 1/Jagged 1 signaling pathways.

HMGB1 is an important chromatin protein that interacts with nucleosomes, transcription factors, and histones to organize DNA and regulate transcription^31^,^32^. HMGB1 can be secreted by immune cells including activated macrophages, acting as a cytokine in response to inflammation^33^. Others have reported that HMGB1 release is induced in airway epithelium and isolated macrophages due to intranasal LPS and hyperoxia as well as hyperoxia exposure alone^28^,^34^. HMGB1 is a ligand for the Receptor for Advanced Glycation End Products (RAGE) and Toll-like Receptors (TLRs) which upon activation further propagate inflammation through NFκB-mediated mechanisms. Our data indicate modest, yet statistical increases in HMGB1 levels in the media of macrophages exposed O_2_ and/or LPS and these increases are attenuated by DHA supplementation *in vivo* (Fig. 1f). A similar result was observed with LPS treatment in macrophages exposed to a short term DHA supplementation *in vitro* (Fig. 2f) but no effect of O_2_ was observed. Whether DHA blocked HMGB1 release through passively decreasing cell death or actively by blocking secretory lysosomal release is beyond the scope of these studies^33^,^35^. Our data implicate that DHA was able to block the release of HMGB1 and we speculate that this blockage may be important in protecting cells from further injury through activation of RAGE or TLRs.

Co-culture studies addressed the effects of activated macrophages on adjacent cells, specifically lung epithelial cells^21^. Our data demonstrate that epithelial cells co-cultured with macrophages previously exposed to O_2_/ LPS release increased levels of HMGB1. This indicates that epithelial cells can be injured by mediators released from O_2_/LPS-activated macrophages. Supplementation with DHA *in vivo* only attenuated this release at the 24 h time point (Fig. 3a). A similar responses was observed in the cells supplemented with DHA *in vitro* at 24 hours but this attenuation was not statistically significant (Fig. 3b).

Leukotriene B_4_ (LTB_4_) is released by many cell types including macrophages in response to inflammation^21^,^36^. Increases in extracellular LTB_4_ are responsible for neutrophil recruitment and potentially trans-epithelial migration of leukocytes to the site of injury^37^. LTB_4_ release from the epithelial cells was elevated by co-culture with macrophages previously exposed to O_2_ and/or LPS. This elevation in LTB_4_ was prevented when the macrophages were supplemented with DHA, specifically *in vitro*, however no differences were observed in cells isolated from mice supplemented with DHA *in vivo* (Fig. 3b,d). These differential responses may be due to the acute verses chronic exposure to DHA and may be linked to formation of anti-inflammatory lipid mediators verses incorporation of DHA into cell membranes and changes in receptor responses, respectively. To further characterize injury in the epithelial cells, flow cytometry was performed to assess apoptosis and cell growth. MLE12 cells demonstrated modest changes in cl-caspase 3 and Ki67 expression in response to co-culture with macrophages exposed to O_2_ and/or LPS however, DHA supplementation attenuated these responses (Fig. 4).

Primary macrophage cultures are not necessarily pure macrophages. To verify our findings in a more pure macrophage cell population, we repeated the co-culture studies using a mouse macrophage cell line, MHS cells. After supplementation with DHA, MSH cells were exposed to O_2_ and/or LPS and co-cultured with MLE12 cells. Responses similar to primary macrophages were observed with increases in HMGB1 and LTB_4_ release (Fig. 5a,b) and increases in cl-caspase 3 and decreases in Ki67 (Fig. 5c,d). These responses were attenuated when the MHS cells were supplemented with DHA.

DHA is an omega-3 fatty acid with proven anti-inflammatory properties^38^. While attenuation of in NFkB activity has been shown in response to DHA supplementation, the mechanisms responsible for decreases in inflammation have not yet been completely deciphered^4^. DHA has also been demonstrated to change membrane properties, specifically membrane fluidity and lipid raft composition^6^. Currently, we demonstrate that dietary DHA supplementation for two weeks prior to macrophage isolation was able to dramatically suppress Notch 1 and Jagged 1 protein expression in response to O_2_ and LPS. This finding was not recapitulated in macrophages isolated from mice fed standard chow and exposed to DHA for 24 h *in vitro*. We did however observe decreases in caspase 9 expression and HMGB1 release in response to DHA supplementation *in vivo* or *in vitro*.

These data would imply that incorporation of DHA into the cell membrane, as would take place over time with dietary supplementation, is responsible for the changes in Notch 1/Jagged 1 expression. Future studies will explore the changes in key canonical Notch1 regulated proteins to determine the functional significance of our findings. While the effects of DHA on apoptosis in the primary macrophages or in co-culture with epithelial cells was evident, the mechanisms for this response are not straightforward and may be a result of decreased Notch signaling and BCL2 pro-apoptotic responses or may be a result of changes in RAGE and TLR activation due to decreases in the ligand, HMGB1. These investigations provide support for the hypothesis that DHA anti-inflammatory affects are in part through altered membrane physiology and receptor expression.

## Materials and Methods

### Animals and Exposure

All animal experiments were performed after approval by the Institutional Animal Care and Use Committee (IACUC) at The Research Institute at Nationwide Children’s Hospital, Columbus, OH and carried out in accordance with the approved guidelines. Equal numbers of male and female C3H/HeN mice (n = 12) were fed standard diets or placed on control diets (CD) or DHA supplemented diets (DHA) as described previously^39^. CD and DHA diets contained equal but enhanced amounts of omega-3 fatty acids within a purified diet base. The CD contained linolenic acid as the only source of omega-3 fatty acids while the DHA diets contained a mixture of linolenic and DHA. The amount of DHA consumed by the mice fed DHA supplemented diets was approximately 63 mg/day. Macrophages were isolated from mice fed standard diets for the studies with *in vitro* supplementation to prevent any confounding effects of endogenous synthesis of DHA from the increased amounts of linolenic acid.

After two weeks, mice were anesthetized by ketamine/xylazine overdose and the lungs were perfused with 10 ml heparinized PBS (≥ 15 units/10 ml heparin, Sigma-Aldrich) by direct injection into the heart. The perfused lungs were excised and the lungs placed in tissue culture media comprised of c-DMEM (Gibco Life Technologies, Grand Island, NY) and 0.7% collagenase/0.03% DNase (Sigma Chemical Co., St. Louis, MO).

### Lung Macrophage Isolation

#### Digestion

Lungs, collected in cDMEM and collagenase/DNase, were cut into small pieces (~1–2 mm^2^) using sterile razor blades. The pieces were incubated in in collagenase/DNase solution at 37 °C for 40 minutes to allow for complete digestion of the tissues. Remaining small pieces were crushed using Cell dissociation sieve - tissue grinder kit. To remove red blood cells, the single cell suspensions were centrifuged at 1200 rpm for 5 min at 4 °C. Finally, 10 ml of c-DMEM was added to the suspension and the mixture was centrifuged for 5 min at 4 °C. The supernatant was discarded and cells were suspended in 1 ml c-DMEM and counted.

#### Staining

One × 10^6^ cells were used for staining. The cells were blocked in 50 μl of Fc (fragment crystelizable) block solution containing 1 μl of CD16/32 blocking antibody in 2 ml of stain wash buffer (SWB, 1% sodium azide, 2% FBS in PBS). The cells were incubated on ice for 10 min, centrifuged for 5 min at 4 °C, washed with SWB and stained with Mac-3 antibody for 30 min in dark (3 μl/1 × 10^6^ cells)(BioLegend, San Diego, CA). Finally, the cells were washed two additional times and suspended in 1 ml SWB.

#### Macrophage sorting by FACS

The above stained cells were separated flow cytometry. Ten-15% of the total cell population was found to be Mac-3 positive and these cells were used for further culture and experimentation.

### Cell culture and treatment

Lung primary macrophages were cultured in DMEM with 4.5 g/L-glucose, 10% FBS, and 1% penicillin/streptomycin (5000 IU/ml) (Cellgro, Mediatech, Inc., Manassas, VA) at 37 °C and 5% CO_2_. Cells were plated in 6 well plates and at a density of 2 × 10^6^ cells/well for western blots and co-culture experiments and 1 × 10^6^ cells/well for for all other measurements. Macrophages isolated from mice fed standard diets were split and half were incubated in media supplemented with DHA (20 μM) while the remainder were incubated in standard media (Supplemental Figure 1). Macrophages isolated from mice fed control diet or DHA supplemented diet were plated independently. After overnight culture, the macrophages were placed in 85% oxygen (O_2_) for a total of 24 h. During the 24 h O_2_ exposure, cells were also treated with LPS (10 ng/ml) for either 6 and 24. This low dose was chosen to mimic the subtle inflammatory responses observed in our mouse model (Supplemental Figure 1). The treatments were timed such that all treatments and exposures terminated at same time.

### Primary macrophage/MLE12 and MSH/MLE12 co-culture

For co-culture experiments, primary macrophages isolated from mice fed standard diet, control diet, or DHA supplemented diet were plated in six well co-culture inserts (Falcon’s Transparent PET Membrane, 2.0 μm pore size, 1 × 10^5^ pores/cm^2^). The macrophages cultured in the transwell inserts were treated as described above, washed with fresh media, and placed over wells which contained confluent MLE12 cells (1 × 10^5^ cells/well) in HITES media, described elsewhere^40^. The procedure was using an immortalized mouse alveolar macrophage cells line (MHS). MHS cells were cultured in RPMI-1640 (ATCC, Manassas, VA) under standard conditions. After 24 h of growth, the culture media was harvested from individual wells and the levels of HMGB1 and LTB_4_ were measured (MHS cytokine responses, Supplemental Figure 1). The MLE12 cells were stained and evaluated for apoptosis, cl-caspase 3, and proliferation, Ki67, by flow cytometry.

### Enzyme-linked immunosorbent assay (ELISA)

Culture media from co-culture experiment, specifically the lower compartment containing MLE12 cells, was assessed for LTB_4_ levels using Cayman Express ELISA Kit (Cayman chemicals company, Michigan, USA).

### Glutathione pathway measurements

Total macrophage cell lysates were prepared in cell lysis buffer with triton and protease inhibitors (okadaic acid, aprotonin, PMSF and leupeptin) and protein concentrations were determined by Bradford assay (Bio-Rad, Hercules, CA). Glutathione (GSH) and glutathione disulfide (GSSG) were measured by the enzyme recycling method as previously described^41^. Gluathione peroxidase (GPx) and glutathione reductase (GR) levels were measured by the methods as described elsewhere^42^.

### Western blot

Total macrophage cell lysates were prepared in SDS sample buffer. For assessment of extra-cellular high-mobility group protein B1 (HMGB1), cell media was precipitated using 40% TCA (1:4) overnight at 4 °C and the protein pellet washed and dissolved in SDS sample buffer. The proteins separated by SDS-PAGE were transferred to nitrocellulose membranes and probed with anti-human rabbit monoclonal antibodies (dilution, 1:500) targeting Notch1, Notch2, Jagged 1, DLL3 (Cell Signaling Technology, Inc., Danvers, MA), caspase-9, and HMGB1 (Abcam, Cambridge, MA). Protein loading was normalized to b-actin using a mouse monoclonal antibody (1:5,000) (Abcam, Cambridge, MA). Membranes were subsequently probed with species specific secondary antibodies for 1 h at room temperature. Bands were visualized using Amersham ECL Prime^®^ Western Blotting Detection Reagent (GE Healthcare, Buckinghamshire, UK) and the band intensity was measured by densitometry.

### Flow Cytometry

MLE12 cells co-cultured with exposed primary macrophages were processed for flow cytometry to estimate apoptosis (cl-caspase 3) or cell growth (Ki67). Cell were fixed as described previously (primary macrophage sorting) and stained with anti-rabbit cleaved caspase-3 primary antibody followed by Alexa Fluor^®^ 647 conjugated IgG secondary antibody (1:500) or Ki-67 Alexa Fluor^®^ 488 conjugated (1:200 dilution) (Cell Signaling Technology, Inc., Danvers, MA) primary antibody. Cells were incubated for 30 min in dark on ice followed by centrifugation and washing with SWB 2 times. The stained cells were analyzed using flow cytometry (BD biosciences, Franklin Lakes, NJ). Final calculations were performed using FlowJo software (Flow Jo LLC, Ashland, OR).

### Statistics

Data were analyzed using Multivariate or Univariate Linear Regression Models with diet as a fixed factor and treatment and exposure as co-variants. Two and 3-way interactions were assessed using between subject effects. Related measurements were analyzed together and corrected for multiple analyses. All data sets were analyzed by Levene’s Test of Equality of Error Variances to determine distribution. If Levene’s Test revealed that data were unevenly distributed then that data were transformed into natural log (ln). Effects and interactions were noted on the individual graphs. Tukey’s post hoc analyses were performed to identify individual difference and were noted by symbol; * different than CD (vehicle)-RA/PBS; ^#^ different than same treatment (difference between diets).

## Additional Information

**How to cite this article**: Ali, M. *et al*. DHA Suppresses Primary Macrophage Inflammatory Responses via Notch 1/ Jagged 1 Signaling. *Sci. Rep*. **6**, 22276; doi: 10.1038/srep22276 (2016).

## Supplementary Material

Supplementary Information

## Figures and Tables

**Table 1:** Glutathione related antioxidants.

	GSH *(nmol/mg pro)*			
	*in vivo*		*in vitro*	
	CD	DHA diet	control	DHA
RA/PBS	17.9 ± 0.5	22.1 ± 0.3*#	17.9 ± 0.5*	43.9 ± 0.9*#
RA/LPS	27.8 ± 1.3*	28.1 ± 0.6*	38.3 ± 0.4*	31.6 ± 1.4*
O_2_/PBS	33.4 ± 0.7*	26.8 ± 0.2*#	32.8 ± 0.6*	36.5 ± 0.5*
O_2_/LPS(6h)	32.2 ± 0.06*	31.6 ± 0.6*	32.2 ± 0.6*	27.9 ± 0.6*#
O_2_/LPS(24h)	26.0 ± 0.6*	32.1 ± 0.1*#	28.2 ± 0.4*	27.2 ± 0.1*
	*effect of DHA, LPS, O*_*2*_interactions between DHA*LPS, DHA O_*2*_, LPS*O_*2*_,DHA*LPS*O_*2*_		*effect of DHA, LPS, O*_*2*_interactions between DHA*LPS, DHA O_*2*_, LPS*O_*2*_,DHA*LPS*O_*2*_	
	**GSSG *(nmol/mg pro)***			
RA/PBS	6.0 ± 0.7	8.0 ± 0.4	6.0 ± 0.7	18.4 ± 0.8*#
RA/LPS	8.9 ± 0.7*	10.2 ± 0.4*	17.0 ± 0.1*	13.4 ± 0.8*
O_2_/PBS	11.1 ± 0.9*	9.3 ± 0.5*	11.1 ± 0.9	10.1 ± 2.8
O_2_/LPS(6h)	13.3 ± 0.3*	10.5 ± 0.4*#	13.2 ± 0.3*	12.3 ± 0.2*
O_2_/LPS(24h)	9.8 ± 0.5*	11.3 ± 0.1*	9.5 ± 0.5	10.1 ± 0.1
	*effect of LPS, O*_*2*_		*effect of LPS, O*_*2*_	
	**GR (μ*mol/min/mg pro)***			
RA/PBS	4.3 ± 0.2	21.3 ± 1.7*#	4.3 ± 0.2	46.5 ± 2.7*#
RA/LPS	1.7 ± 0.7	35.5 ± 1.4*#	46.0 ± 7.1*	25.7 ± 1.5*
O_2_/PBS	32.1 ± 0.9*	34.8 ± 0.1*	32.1 ± 0.9*	37.4 ± 2.1*
O_2_/LPS(6h)	41.2 ± 4.5*	33.2 ± 0.4*	12.7 ± 0.7	14.3 ± 1.6
O_2_/LPS(24h)	31.1 ± 5.9*	45.6 ± 1.8*#	22.8 ± 2.1*	23.2 ± 0.7*
	*effect of LPS, O*_*2*_		*effect of LPS, O*_*2*_	
	**GPx (μ*mol/min/mg pro)***			
RA/PBS	69.3 ± 19.0	69.3 ± 19.0	62.2 ± 18.2	34.9 ± 14.1
RA/LPS	97.7 ± 29.4	110.5 ± 29.6	82.2 ± 21.0	98.1 ± 24.7
O_2_/PBS	44.3 ± 20.8	44.3 ± 20.9	83.7 ± 21.1	95.0 ± 28.4
O_2_/LPS(6h)	86.8 ± 23.7	78.5 ± 19.9	51.4 ± 13.1	70.4 ± 17.7
O_2_/LPS(24h)	61.8 ± 21.1	122.5 ± 46.0	74.8 ± 19.1	103.4 ± 26.3
	*no differences*		*no differences*	

Members of the glutathione antioxidant system were measured in isolated macrophages as described in Methods. CD: isolated from mice fed control diet, *in vivo*; DHA diet: isolated from mice fed a DHA supplemented diet, *in vivo*; control: isolated from mice fed standard diet and treated with vehicle, *in vitro*; DHA: isolated from mice fed standard diet and treated with DHA, *in vitro*. The data reflect n = 3 from three independent experiments. Data were analyzed by Multivariate Linear Regression with Tukey’s post hoc. * indicates different that CD RA/PBS, ^#^ indicates different than same treatment (difference between diets), p < 0.05.
