# Supplementary figures and images for: DHA Suppresses Primary Macrophage Inflammatory Responses via Notch 1/ Jagged 1 Signaling
Source: Sci Rep. 2016 Mar 4;6:22276. doi: 10.1038/srep22276 (PMC4778022; doi:10.1038/srep22276)

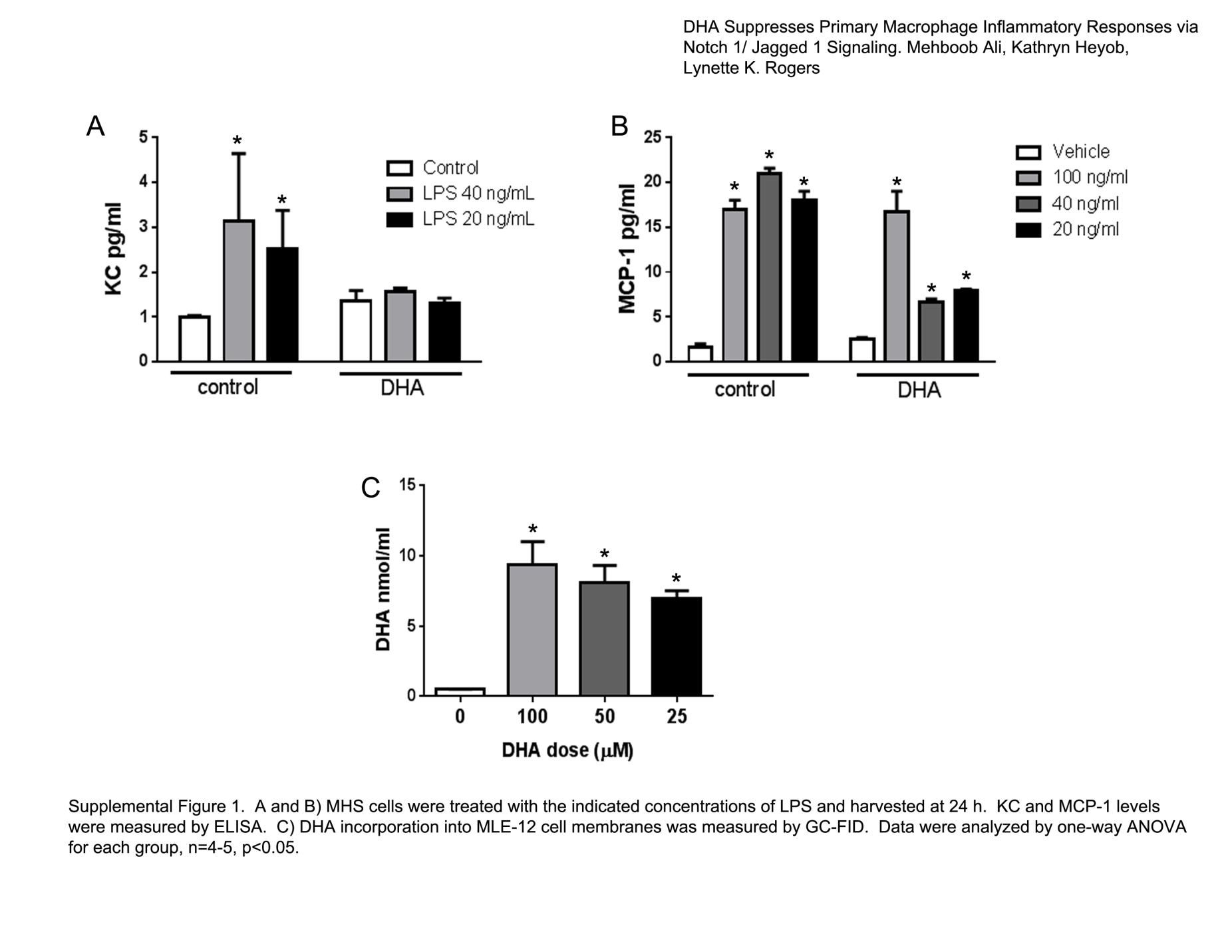


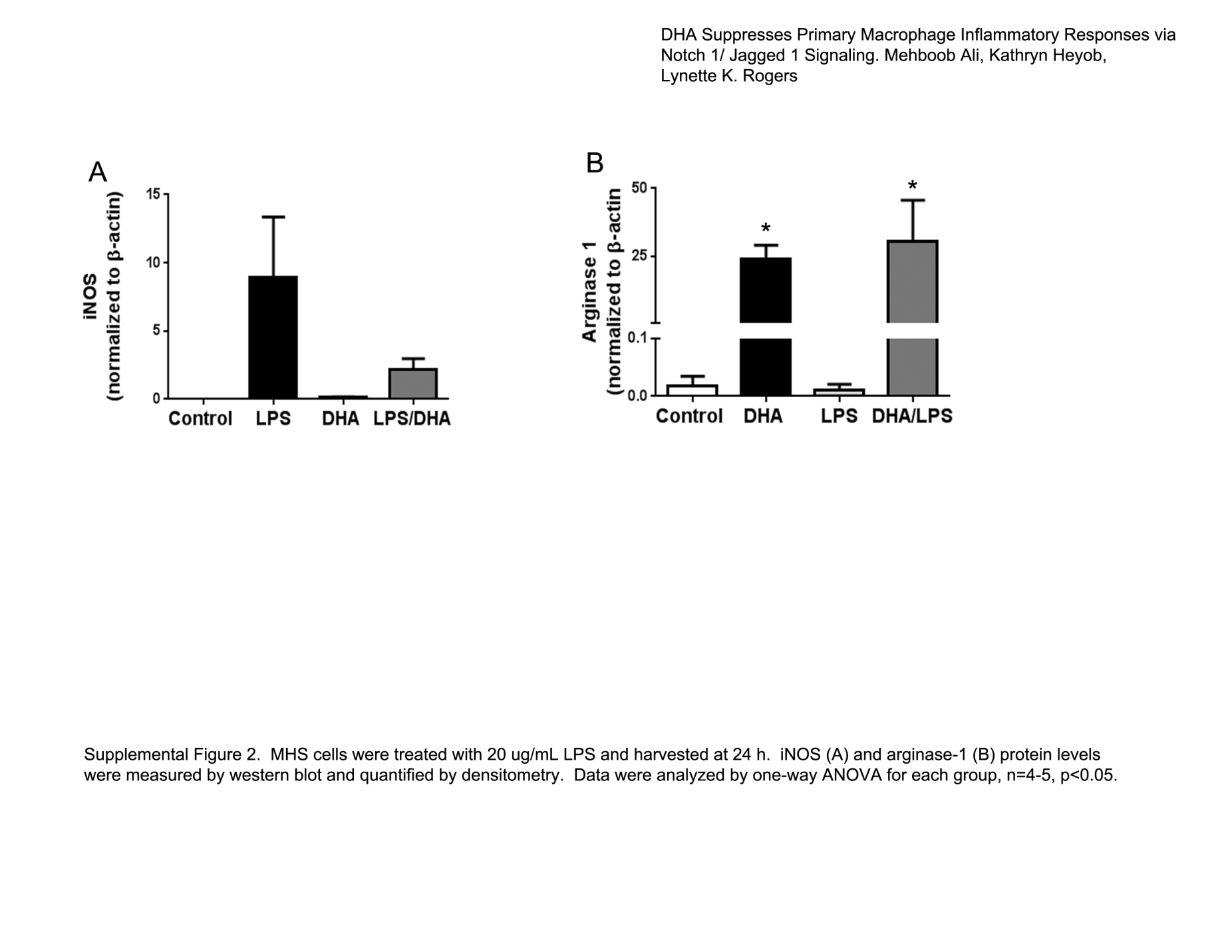

Supplement: Supplementary Information [file srep22276-s1.doc]
